# Addition of Anti-Angiogenetic Therapy with Bevacizumab to Chemo- and Radiotherapy for Leptomeningeal Metastases in Primary Brain Tumors

**DOI:** 10.1371/journal.pone.0155315

**Published:** 2016-06-02

**Authors:** Michael C. Burger, Pia S. Zeiner, Kolja Jahnke, Marlies Wagner, Michel Mittelbronn, Joachim P. Steinbach

**Affiliations:** 1 Dr. Senckenberg Institute of Neurooncology, Goethe University, Frankfurt, Germany; 2 Institute of Neurology (Edinger Institute), Goethe University, Frankfurt, Germany; 3 Department of Neurology, Goethe University, Frankfurt, Germany; 4 Institute of Neuroradiology, Goethe University, Frankfurt, Germany; University Hospital of Navarra, SPAIN

## Abstract

Leptomeningeal dissemination of a primary brain tumor is a condition which is challenging to treat, as it often occurs in rather late disease stages in highly pretreated patients. Its prognosis is dismal and there is still no accepted standard of care. We report here a good clinical effect with a partial response in three out of nine patients and a stable disease with improvement on symptoms in two more patients following systemic anti-angiogenic treatment with bevacizumab (BEV) alone or in combination with chemo- and/or radiotherapy in a series of patients with leptomeningeal dissemination from primary brain tumors (diffuse astrocytoma WHO°II, anaplastic astrocytoma WHO°III, anaplastic oligodendroglioma WHO°III, primitive neuroectodermal tumor and glioblastoma, both WHO°IV). This translated into effective symptom control in five out of nine patients, but only moderate progression-free and overall survival times were reached. Partial responses as assessed by RANO criteria were observed in three patients (each one with anaplastic oligodendroglioma, primitive neuroectodermal tumor and glioblastoma). In these patients progression-free survival (PFS) intervals of 17, 10 and 20 weeks were achieved. In three patients (each one with diffuse astrocytoma, anaplastic astrocytoma and primitive neuroectodermal tumor) stable disease was observed with PFS of 13, 30 and 8 weeks. Another three patients (all with glioblastoma) were primary non-responders and deteriorated rapidly with PFS of 3 to 4 weeks. No severe adverse events were seen. These experiences suggest that the combination of BEV with more conventional therapy schemes with chemo- and/or radiotherapy may be a palliative treatment option for patients with leptomeningeal dissemination of brain tumors.

## Introduction

Leptomeningeal dissemination (LMD) of primary brain tumors such as low-grade and anaplastic gliomas, glioblastomas, medulloblastomas (MB) and primitive neuroectodermal tumors (PNET) is a condition which considerably worsens the prognosis of the underlying disease [1,2]. Its frequency is most likely underestimated as in autopsy series it is found more commonly than in clinical series: while LMD was clinically reported in 2–4% [1,3] of patients with malignant glioma, autopsy studies reported a higher frequency of 6–21% [4,5]. Furthermore the incidence of LMD seems to be increasing over the last decades due to the improvement of both primary therapies and diagnostic means [4]. Spinal and leptomeningeal deposits of glioblastoma, in particular, are mainly recognized in advanced stages [6] and occur more frequently in younger patients [1]. Established therapy regimes include involved-field radiotherapy (IFXRT) of symptomatic lesions with a total dose of 25–40 Gy. This may result in some palliation, in particular of meningeal signs and cauda equina syndromes but its effect is rather vague with a median overall survival (mOS) between 2.8 and 16.8 months and no effect on median progression-free survival (mPFS) [5,7,8]. Furthermore, in patients with nerve compression or radicular syndromes like radicular pain due to compression of nerval structures in bony nerve channels caused by LMD, involved-field radiation therapy (IFXRT) and whole brain radiation therapy (WBXRT) have a good palliative effect [9]. Intrathecal therapy with methotrexate, cytosine arabinoside, Depocyt (cytarabine liposome) or thioTEPA has been used in LMD, with only modest differences in respect to toxicity and efficacy [10,11]. Published series report median progression-free survival (mPFS) intervals between 3.0 and 4.9 months and median overall-survival (mOS) intervals between 3.5 and 11.3 months for patients with LMD from malignant gliomas [1,7,12]. These diverging reports could be explained by varying timepoints of diagnosis of LMD, pretreatment and histology of the underlying primary brain tumor as well as the different therapy schemes used. On intrathecal administration of Depocyt in LMD of malignant glioma, only three out of nine patients received at least six weeks of treatment before further progression and the longest duration of treatment was 14 weeks [7]. Comparable to the multimodal treatment strategies for primary brain tumors in general, combinatorial approaches (i.e. combining radiotherapy with systemic or intrathecal chemotherapy) seem to be most promising [1,12]. However, intrathecal chemotherapy penetrates only few millimeters into brain tissue or subarachnoideal tumor nodules and therefore is not sufficient for the common nodular subtype of LMD [13]. Established systemic chemotherapies like temozolomide (TMZ) or alkylator regimens can be potent, however many patients have already received extensive pretreatment and therefore further chemotherapeutic treatment may not be highly promising. The rationale for combining anti-angiogenic therapies, e.g. bevacizumab (BEV) with these established therapy regimes in leptomeningeal gliomatosis is unclear, since often a significant portion of the malignant cells is dispersed in the cerebrospinal fluid (CSF). For this reason, anti-angiogenic strategies at first look do not seem to be purposeful. On the other hand, high VEGF levels in the CSF are known as a negative prognostic marker in LMD of solid tumors [14]. Increased proliferation through autocrine VEGF signalling under cell culture conditions has been described both for glioblastoma and medulloblastoma cells [15,16]. Further, clinical symptoms mostly arise from the compression of nerve roots or the spinal cord by tumor nodules or infiltration of these structures. While small nodular metastases and a diffuse “sugarcoating” of superficial neural structure can be supplied with nutrients solely by convection from the CSF, for the process of growth of leptomeningeal metastases beyond a certain size neo-angiogenesis is an essential step [17]. Therefore adding anti-angiogenic therapies like BEV may bring further benefit. BEV may alleviate edema and compression of cranial nerves, the spinal cord and nerve roots, by normalizing leaky blood-brain-barrier function.

For glioblastoma, one case report of a patient with combined intramedullary and leptomeningeal spread treated with radiotherapy and BEV monotherapy exists [18]. In this patient stable disease (SD) was achieved with a progression-free survival (PFS) of three months and an overvall survival (OS) of six months. Bae et al. [19] included in their patient series three patients with LMD treated with intrathecal methotrexate (MTX) additionally to systemic BEV and irinotecan. Two of those patients suffered from secondary glioblastoma, the third from pleomorphic xanthoastrocytoma with atypical features. OS after dissemination was 2, 5 and 2 months, respectively. While there is no such case report for primitive neuroectodermal tumor (PNET), one case report included one adult patient with recurrent medulloblastoma and LMD treated with BEV [20]. In this patient, monotherapy with BEV resulted in complete response (CR) with a progression-free survival (PFS) of almost 17 months, after which the patient died from an unrelated cause.

Bevacizumab has been used repeatedly in our center for the treatment of LMD of primary brain tumors, mostly in combination with chemo- and/or radiotherapy. However, the scientific knowledge of the benefits of bevacizumab in this situation is still very limited and up to date no case series focusing on the use of BEV in patients with LMD from brain tumors exist. Therefore, we performed a retrospective data analysis of all patients with LMD from primary brain tumors treated with BEV alone or in combination at our institution.

## Patients and Methods

We report on nine consecutive patients treated during a time period of seven years between July 2008 and June 2015 with BEV (10mg/kg IV every 2 weeks) in combination with lomustine (CCNU), temozolomide (TMZ), irinotecan and/or radiotherapy or as a single agent on an individual basis. One patient suffered from an astrocytoma WHO°II, one from an anaplastic astrocytoma WHO°III, one from an anaplastic oligodendroglioma WHO°III, two patients from a primitive neuroectodermal tumor (PNET) WHO°IV and four patients from a glioblastoma WHO°IV (see Table 1). During the observation period, we treated approximately 264 patients with glioma WHO°II-III, 7 patients with PNET WHO°IV and 701 patients with glioblastoma (GB) WHO°IV in our center. In 6 patients (2.3%) with glioma WHO°II-III, 2 patients (28.6%) with PNET WHO°IV and 25 patients (3.6%) with GB WHO°IV the diagnosis of symptomatic LMD was established. These rates with a rather low incidence of LMD in gliomas WHO°II-III, a high incidence in PNET and a medium incidence in GB are in accordance with published data [2,7]. The decision for anti-angiogenic therapy with bevacizumab was subjected to a negative and positive selection. Patients with a Karnofsky patient score of ≤50% were usually given a recommendation for best supportive care (BSC), and patients with limited alkylator pretreatment were usually treated with a chemotherapy based exclusively on temozolomide or lomustine (CCNU). As the effect of bevacizumab on overall survival (OS) is very controversial [21,22], the decision for therapy with bevacizumab was restricted to (I) patients with extensive alkylator pretreatment, (II) patients with relevant myelosuppression (thrombocytes < 100/nl or leukocytes < 3/nl) and (III) patients with ongoing or (IV) anticipated clinical deterioration (based on imaging results) where an alkylator monotherapy was deemed insufficient by the treating physician (see Table 1). The decision in favour of combination chemotherapy was based on prior treatment and discussed openly with the patients. Given the fact that all patients with exception of patients 1 and 8 have received involved-field radiation therapy (IFXRT) of the brain before, whole brain radiation therapy (WBXRT) was deferred if possible. Rather, the patients received IFXRT of clinically symptomatic areas. In none of the patients LMD was already present at initial diagnosis, typically the diagnosis was established late in the course of the disease (see Table 1), which is consistent with other published reports [23]. The diagnosis of LMD was based both on leptomeningeal enhancement in magnetic resonance imaging (MRI) scans and CSF analysis. Since none of the patients of our series had an Ommaya reservoir implanted, CSF was taken by lumbar puncture. In all but one patient (patient 6) malignant cells were verified by CSF cytology. In patient 6, suspicious cells were seen which did not fulfil the diagnostic criteria for atypical neoplastic cells and were negative for GFAP (glial fibrillary acid protein). However, diagnosis was established on the basis of indicative imaging in combination with typical CSF parameters (cell number 101/μl, total protein 14 g/dl, lactate 8.45mmol/l, CSF glucose 20.1mg/dl). All nine patients showed a mix of diffuse and nodular leptomeningeal dissemination on imaging. Contrast-enhanced MRI scans were done at intervals of four to eight weeks and assessed according to RANO criteria [24].

**Table 1:** Patient characteristics.

Pat. No.	Age	Gender	Primary tumor localisation	Histology	Pretreatment	Decision criteria for BEV therapy	Time between diagnosis and evidence of LMD [months]
1	35	M	intra-medullar Th11	A	S, XRT-TMZ, TMZ, Re-XRT, CCNU	I, III	45
2	31	M	thalamus	AA	XRT-TMZ, TMZ, CCNU, Depocyt	I, II*, III	13
3	33	M	left frontal	AO	S, TMZ, Re-S, XRT	IV	10
4	35	M	pontine	PNET	XRT, CCV	III	14
5	56	M	bifrontal	PNET	S, XRT-TMZ, TMZ	III	9
6	37	F	left frontal	GB	S, XRT-TMZ, TMZ	III	25
7	44	F	right parietal	GB	S, XRT-TMZ, TMZ	III	7
8	46	M	gliomatosis cerebri	GB	PC, CCNU, TMZ	I	28
9	55	M	left temporal	GB	S, XRT-TMZ, TMZ	IV	7

I = extensive alkylator pretreatment; II = myelosuppression; III = ongoing deterioriation; IV = anticipated clinical deterioration;

* = thrombocyte nadir of 20/nl;

A = Diffuse Astrocytoma WHO°II; AA = Anaplastic Astrocytoma WHO°III; AO = Anaplastic Oligodendroglioma WHO°III; CCNU = lomustine; CCV = lomustine/cisplatin/vincristine; Depocyt = intrathecal Depocyt; F = female; GB = Glioblastoma WHO°IV; LMD = leptomeningeal dissemination; M = male; ND = not determined; PC = procarbacine/lomustine; PNET = primitive neuroectodermal tumor WHO°IV; Re-XRT repeat involved field radiation therapy; Re-S = repeat surgery; S = surgery; TMZ = temozolomide 5/28; XRT = involved field radiation therapy; XRT-TMZ = involved field radiation therapy with concomitant temozolomide.

Our institutional review board approved this retrospective study and patients gave their written consent for scientific work with clinical data including MRI scans (ethics committee at the University Hospital Frankfurt; reference number 04/09-SNO 01/09).

## Results

**Fig 1:**
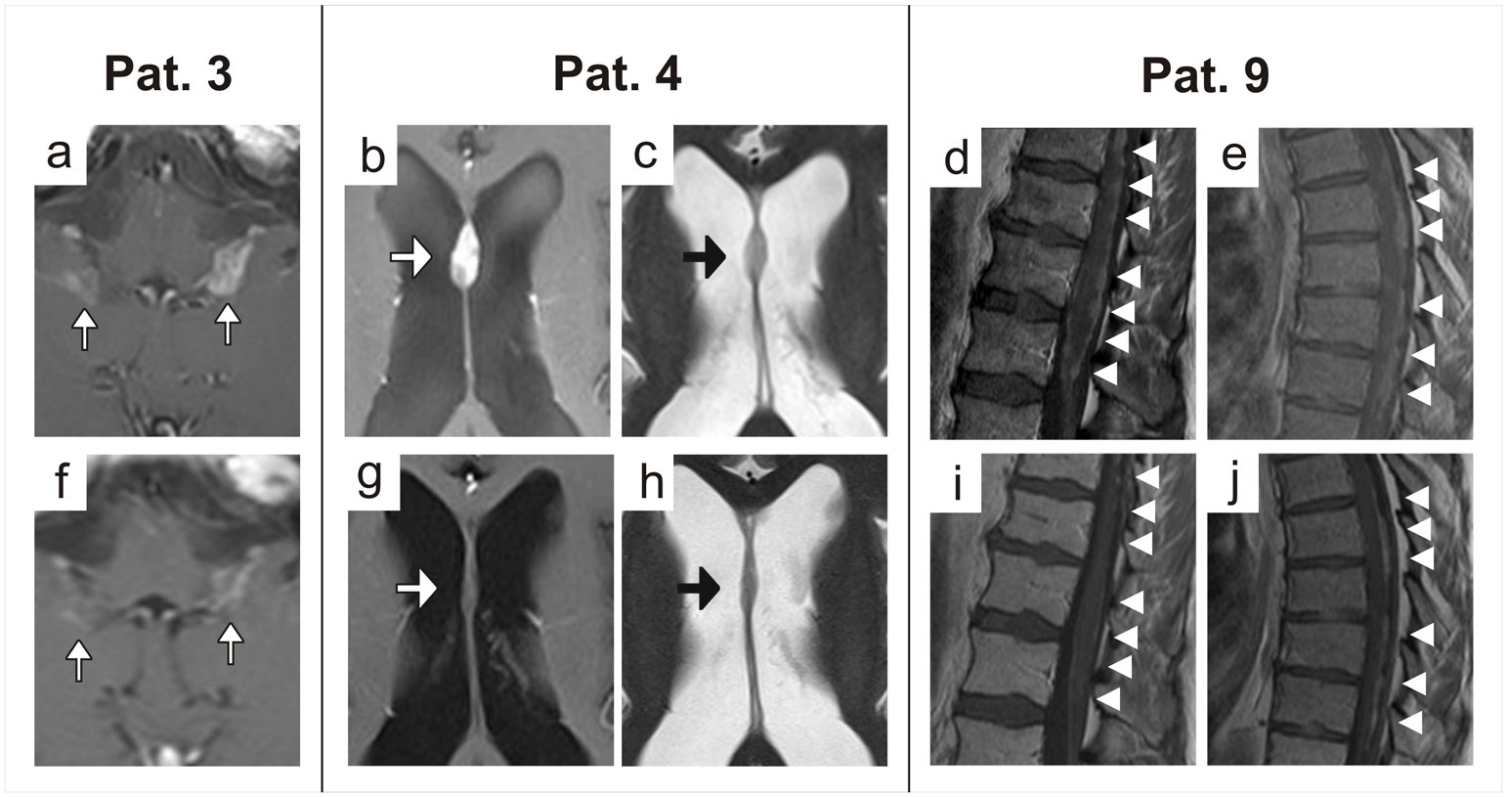
MRI scans before (a-e) and under (f-j) therapy. **a, f:** Reduced leptomeningeal enhancement (white arrows) after 8 weeks of therapy with bevacizumab and lomustine in patient 3. **b, g:** Regression of leptomeningeal contrast-enhancing nodule (white arrow) on the septum pellucidum on T1-weighted images after eight weeks of therapy with bevacizumab and temozolomide in patient 4. **c, h:** This regression (black arrow) in patient 3 was also visible on T2-weighted images, which makes pure pseudoresponse unlikely. **d, i:** Regression of leptomeningeal contrast-enhancing nodules (white arrowheads) on the surface of the medullar conus and the lumbar nerve roots on T1 weighted images (Th10-L2) in patient 9 before and after radiotherapy plus eight weeks of therapy with bevacizumab and lomustine. **e, j:** This regression of contrast-enhancement (white arrowheads) in patient 9 was also apparent in the thoracic spine (Th5-Th9) which was not treated with radiotherapy.

In patients 3, 4 and 9 rapid, impressive and sustained regression of nodular leptomeningeal contrast-enhancing lesions was achieved (partial response, PR). Contrast enhancing nodules considerably shrank and there was also some improvement of diffuse leptomeningeal enhancement (Fig 1a–1e and 1f–1j). Improvement was apparent in T2-weighted sequences as well. Pure pseudoregression through normalisation of the blood brain barrier therefore seems unlikely. Notably, in patient 9, regression of contrast enhancing lesions was observed beyond the field of involved-field radiotherapy (IFXRT; Fig 1e and 1j), therefore this effect cannot be explained by the radiotherapy but most likely is an effect of BEV therapy. Furthermore, the patient´s clinical condition improved clearly. An impressive clinical and radiological improvement was also observed in patients 3 and 4 (see Tables 2 and 3). However, as these patients received additional therapy in the form of involved-field radiotherapy (IFXRT) of the area affected or dose dense temozolomide chemotherapy, the determining factor for the observed response is not entirely clear. In patients 3, 4 and 9 progression-free survival (PFS) intervals of 17, 10 and 20 weeks and overall survival (OS) intervals of 20, 17 and 24 weeks respectively were achieved. In patients 1, 2 and 5 stable disease (SD) was reached. While patients 1 and 2 clinically improved, symptoms were stabilised in patient 5. In these three patients PFS intervals of 13, 30 and 8 weeks respectively were reached. In patients 1 and 5 OS intervals of 23 and 14 weeks were observed, whilst patient 2 is still alive. Patient 2 was initially a non-responder to a combination therapy with CCNU per os and Depocyt intrathecally over eight weeks (delate clinically and radiographically). After changing to monotherapy with BEV, clinical deterioration was halted and stabilisation of disease was achieved radiographically (see Table 2). Patients 5, 6 and 7 progressively deteriorated and the first imaging revealed progressive disease (PD). These patients had a PFS of 3, 4 and 4 weeks and an OS of 14, 11 and 11 weeks respectively. Summarized, the median progress-free survival (mPFS) of all nine patients was 10 weeks and the median overall-survival (mOS) was 15.5 weeks. In those five patients in which surveillance sampling of CSF was performed (patients 1–5), normalisation of CSF parameters was seen with a reduction of CSF cell number and/or protein concentration. Remarkably, clearing of malignant cells was attained in two out of five patients (patients 4 and 5). Additionally, in patient 2 an initially increased CSF pressure was normalised, which probably contributed to the marked clinical improvement observed (see Table 3). Five patients had clinical signs for raised CSF pressure at start of treatment. During treatment these symptoms disappeared in all patients, except for patient 6 (the only patient evidencing imaging compatible with malresorptive hydrocephalus). In all patients where steroid intake was necessary at start of treatment, a dose reduction was feasible (see Table 3). All patients tolerated BEV treatment well without suffering from typical side-effects like arterial hypertension or proteinuria. Two patients (patients 1 and 3) developed a common toxicity criteria (CTC) grade 3 leukopenia which spontaneously resolved. We attributed this side-effect to the simultaneous chemotherapies with CCNU or TMZ respectively.

**Table 2:** Treatment and outcome.

Pat. No.	Histology	Number of BEV cycles (10mg/kg every 2 weeks)	Simultaneous chemotherapy				Simultaneous radiotherapy			Best response (RANO criteria)	PFS [weeks]	OS [weeks]
			Substance	Dose [mg/m^2^]	Number of cycles	Length of each cycle [weeks]	Target area	Dose	Fractionating scheme			
1	A	6	CCNU	90	4	6	WBXRT; IFXRT Th10 –Cauda equina	40 Gy; 20 Gy	20x 2 Gy; 5x 4 Gy	SD	13	23
2	AA	15	-	-	-	-	-	-	-	SD	30	NR
3	AO	8	CCNU	110	3	6	IFXRT posterior fossa	36 Gy	12x3 Gy	PR	17	20
4	PNET	5	TMZ 7/14	100	5	2	-	-	-	PR	10	17
5	PNET	4	CCNU	90	1	6	-	-	-	SD	8	14
6	GB	2	-	-	-	-	IFXRT posterior fossa and IFXRT C1 –Th3	35 Gy	each 10x 3.5 Gy	PD	3	14
7	GB	2	CCNU	90	1	6	IFXRT posterior fossa—Th1 and IFXRT Th11	36 Gy	each 12x 3 Gy	PD	4	11
8	GB	2	IRI	125	2	2	-	-	-	PD	4	11
9	GB	10	CCNU	90	4	6	IFXRT Th11 –S1	36 Gy	12x 3 Gy	PR	20	24

A = Diffuse Astrocytoma WHO°II; AA = Anaplastic Astrocytoma WHO°III; AO = Anaplastic Oligodendroglioma WHO°III; BEV = bevacizumab; CCNU = lomustine; GB = Glioblastoma WHO°IV; IFXRT = involved field radiation therapy; IRI = irinotecan; NR = not reached; OS = overall survival; PD = progressive disease; PFS = progression free survival; PNET = primitive neuroectodermal tumor WHO°IV; PR = partial response; SD = stable disease; TMZ 7/14 = dose dense temozolomide (one week on / one week off); WBXRT = whole brain radiation therapy.

**Table 3:** Clinical course.

Pat. No.	Presenting symptoms of LMD	Distribution of LMD	Clinical signs of raised intracranial pressure		Imaging compatible with hydrocephalus malresorptivus		Karnofsky patient score (KPS)		Steroid intake [mg of dexamethasone per day]	
			at start of therapy	under therapy	at start of therapy	under therapy	at start of therapy	development under therapy	at start of therapy	under therapy
1	headache	cerebral, spinal	+	-	-	-	70	+10	0	0
2	headache, seizures	cerebral, spinal	+	-	-	-	80	+20	4	0
3	headache, nausea	cerebral, spinal	+	-	-	-	90	+10	8	2
4	headache, seizures, nausea, vomiting	cerebral, spinal	-	-	-	-	60	+20	0	0
5	sensory loss	cerebral, spinal	-	-	-	-	70	±0	4	0
6	headache, pollacisuria	cerebral, spinal	+	+	+	ND	60	+10	16	12
7	paresis, radicular pain	cerebral, spinal	-	-	-	-	60	-10	12	8
8	paresis, radicular pain, sensory loss	cerebral, spinal	-	-	-	-	80	-20	12	6
9	sensory loss, pollacisuria	spinal	-	-	-	-	80	+10	12	0

**+** = present; **-** = not present; LMD = leptomeningeal dissemination; ND = not determined.

## Discussion

In this cohort of patients suffering from LMD from primary brain tumors we report treatment responses to BEV in combination with lomustine, TMZ, irinotecan and/or radiotherapy, or as BEV monotherapy. We are able to demonstrate that these therapy strategies have a clinically meaningful response in this setting. In six out of nine patients, temporary symptom relief or stabilisation was seen. Consistent with the known imaging effects of bevacizumab in brain tumors [25], there was an improvement in nodular disease amongst the responders. The reduction of nodular disease indicates that the main mode of action of bevacizumab may be anti-angiogenic in LMD. However, we cannot rule out that inhibition of the direct autocrine effect of VEGF on tumor cells may be involved [15,16]. Since in most patients of our series, BEV was applied as part of a combination therapy, it remains difficult to attribute treatment response to BEV alone or to its use as cotherapy. However, we consider the following observations as rather convincing evidence for a distinct BEV effect: (I) regression of contrast enhancing lesions beyond the field of involved-field radiotherapy in patient 9, (II) the reversion of clinical deterioration by changeover from a CCNU/Depocyt combination therapy to a BEV monotherapy in patient 2 and (III) the normalisation of the initially elevated CSF pressure in the same patient. These effects cannot be attributed to steroids alone, since steroid doses in all steroid-dependent patients could be decremented during the course of therapy. The feasibility of reducing the steroid dose is also of high significance for the spectrum of adverse events suffered by the patients [26]. The reduction of the CSF pressure under BEV monotherapy and the improvement of several initially pathological CSF parameters strongly argue against the notion of BEV just being an “expensive super steroid” [27] in LMD. Patients with glioblastoma (GB) often exhibit a regression of contrast-enhancement accompanied with a reduction of cerebral oedema after the first BEV infusions [28]. However, an increase in hyperintense alterations is typically seen on T2- and FLAIR-sequences in the case of tumor progression [29,30]. This is of special interest for assessing the imaging of patients which achieved partial responses (patients 3, 4 and 9). In those patients regression of contrast-enhancing tumor areas was seen as well as regression of non-enhancing tumor nodules on T2 sequences, which cannot be explained by pure reduction of oedema. Interestingly enough, in patients with GB the treatment seemed to have a worse effect compared to the patients with non-GB primary brain tumors: While in GB patients, three out of four patients were primary non-responders, all patients with non-GB brain tumors in this series (five out of five) at least reached SD. This striking difference may be due to the enhanced aggressiveness or invasiveness of LMD from GB than that from non-GB brain tumors [7]. Still we consider that the patient number in this series is too low and the underlying histologies and the prior treatment modalities are too diverse to answer such a specific question. More reports on patients with LMD of primary brain tumors and BEV therapy have to be compiled to make a statement on the differential effects on particular primary brain tumor entities. The most significant limitation for the interpretation of this case series is that in all but one patient BEV was used as a combination partner to other therapy modalities. This multimodal therapeutic approach was applied due to the advanced disease stages and critical clinical situation of the patients reported on. Despite these unavoidable shortcomings, this series provides the novel reference that the addition of BEV to more established therapy regimens may have a good effect on the symptoms caused by LMD of brain tumors.

Summarized, the addition of BEV to established therapy regimens may represent a novel palliative treatment option for patients with leptomeningeal disseminated primary brain tumors. Similarly, BEV therapy seems to have a good therapeutic effect in brain metastases from NSCLC [31] and a combination of BEV with irinotecan in patients with refractory brain metastases from breast cancer has shown convincing results [32]. However, while there may be a good symptomatic effect, it is more than doubtful if overall survival (OS) can be improved. Furthermore, treatment with BEV is associated with considerable risks like gastrointestinal perforation, venous thromboembolism or intracranial hemorrhages [33]. To accurately determine the benefit of adding anti-angiogenic therapy to chemo- and/or radiotherapy in leptomeningeal dissemination in brain tumors, prospective clinical trials would be needed. However, these are difficult to perform due to the restricted number of patients with this rare complication of a rare disease, the variable pretreatments and therefore high variation in the course of the disease. The only conceivable approach in successfully realising such trials would be through large multi-institutional initiatives.
